# Multiphysics Analysis and Optimization of a Thin-Film Lithium Niobate Phase Modulator for Fiber-Optic Gyroscopes

**DOI:** 10.3390/mi17060751

**Published:** 2026-06-21

**Authors:** Hanyi Zhang, Rong Fan, Yin Cao, Wenxuan Cheng, Yujie Wang, Jianfeng Bao, Lijing Li

**Affiliations:** 1 School of Instrumentation and Optoelectronic Engineering, Beihang University, Beijing 100191, China; by2117118@buaa.edu.cn (H.Z.); fanrong_buaa@outlook.com (R.F.); yinc0901@buaa.edu.cn (Y.C.); cwx2025@buaa.edu.cn (W.C.); wyj13831391657@163.com (Y.W.); 2 School of Physics and Electronic Information Engineering, Ningxia Normal University, Guyuan 756099, China

**Keywords:** lithium niobate on insulator, phase modulator, multiphysics coupling, fiber-optic gyroscope

## Abstract

Lithium niobate on insulator (LNOI) has emerged as a promising platform for compact, low-loss phase modulators. The extant LNOI studies evaluate device performance almost exclusively through the Pockels effect, treating piezoelectric–photoelastic strain and thermo-optic drift as decoupled channels. Crucially, both mechanisms directly perturb the phase bias of a fiber-optic gyroscope (FOG), rendering them indispensable in sensing-oriented design. This work establishes a unified multiphysics model of an X-cut TFLN ridge phase modulator that self-consistently couples the electro-optic, piezoelectric–photoelastic, thermo-optic, and pyroelectric channels. The contributions of the four mechanisms are quantitatively decomposed under realistic FOG operating conditions, and the slab thickness, ridge-top width, and electrode gap are systematically optimized to balance modulation efficiency against environmental robustness. The co-optimization of the ridge geometry and electrode gap design maintains the EO overlap factor near 0.55, while reducing the half-wave voltage requirement. This results in a half-wave voltage length of *V*_π_*L* = 1.65 V·cm at a 4.4 μm electrode gap. The optimized geometry and electrode gap (4.4 μm) are essentially temperature-independent: extracted from the Pockels modulation slope, *V*_π_*L* remains stable at ≈1.65 V·cm (push–pull single-pass; within ~0.3%) across 25~85 °C. Furthermore, an externally imposed substrate temperature rise of 60 K (the upper end of the 25~85 °C FOG operating range) induces a mode-field-weighted thermal residual corresponding to approximately 27% of the Pockels modulation depth at an applied voltage of 5 V. The present study demonstrates that the DC-coupled operation of TFLN sensor-grade modulators is viable across the full FOG temperature range, without dedicated active temperature stabilization, and the residual thermal-bias offset is absorbed by the FOG’s standard closed-loop servo electronics. The results of the study provide quantitative design guidelines for high-performance, environmentally stable TFLN phase modulators in compact FOG systems.

## 1. Introduction

Fiber-optic gyroscopes (FOGs) have been extensively utilized in inertial navigation, attitude control, and precision measurement, due to their inherent advantages, including the absence of moving components, rapid initialization, high accuracy, and exceptional reliability [1]. The advent of unmanned systems, intelligent transportation, and miniaturized industrial sensing has driven a paradigm shift in inertial device development. These devices are undergoing a transition towards smaller form factors, reduced power consumption, and heightened levels of integration. For FOGs, this trend translates into a stringent set of requirements. In addition to a more compact footprint and reduced drive power, sensor-grade operation demands low bias drift, high long-term stability, and accurate scale factors under realistic environmental conditions [2,3].

The phase modulator is the core optoelectronic component that closes the FOG feedback loop, simultaneously providing beam splitting/combining, polarization filtering, and phase modulation [4,5]. Its half-wave voltage, modulation linearity, and environmental stability translate directly into the bias stability, scale-factor error, and long-term accuracy of the gyroscope. An ideal FOG-grade phase modulator therefore combines a low drive voltage, a compact footprint, a linear phase response, and strong robustness against thermal and mechanical perturbations. Conventional bulk lithium niobate (LN) modulators fabricated by titanium in-diffusion or annealed proton exchange offer mature processing and excellent electro-optic performance [6], but their large size, relatively high drive voltage, and long-term bias drift make it increasingly difficult to satisfy the integration and stability demands of next-generation miniaturized FOGs. To address these limitations, integrated phase modulators have been realized on silicon, indium phosphide (InP), and electro-optic polymer platforms [7]. Each of these platforms, however, exhibits intrinsic limitations for sensor-grade use: silicon modulators rely on the plasma-dispersion effect, and are therefore susceptible to nonlinearity, free-carrier absorption, and temperature sensitivity [8]; InP modulators achieve compact integration, but typically require active thermal stabilization for reliable operation [9]; and polymer modulators still suffer from insufficient long-term reliability [10]. Thin-film lithium niobate on insulator (TFLN), by contrast, combines a high refractive-index contrast, strong optical confinement, low propagation loss, and a highly linear Pockels response, making it one of the most promising platforms for high-performance integrated phase modulators, with demonstrated advantages in low-voltage and high-speed modulation [11,12,13,14,15,16].

Beyond the Pockels effect, lithium niobate also exhibits pronounced direct and converse piezoelectric coupling and a significant thermo-optic response: the modulating electric field induces mechanical strain [17] that perturbs the effective index through the photoelastic effect [18], while temperature variation drives a thermo-optic index drift, together with pyroelectric phase bias fluctuations. Bulk LN phase modulators have been studied extensively, and are supported by a mature device and packaging ecosystem [19]. However, the majority of prior work has focused on characterizing or compensating *V*_π_, bias drift, and residual intensity modulation, without establishing a unified multiphysics description of piezoelectric–photoelastic coupling and thermo-optic drift [20]. In high-precision sensing applications such as FOGs, however, the true phase response under simultaneous electrical drive [21], thermal load, and mechanical perturbation is directly tied to bias stability and long-term measurement accuracy. Several studies have examined the impact of temperature variation [22], mechanical vibration, or packaging stress on the output characteristics of bulk modulators [23], and others have addressed the influence of external perturbations on FOG bias stability; yet these analyses commonly treat thermal and stress effects as independent external disturbances, and do not systematically resolve the physical origin and relative weight of each contribution from the joint action of material constitutive behaviors, waveguide confinement, and device geometry [24,25]. The existing literature, therefore, captures the fact that performance varies under perturbation, but provides little quantitative insight into which coupling mechanisms dominate and how each contributes within a realistic device structure [26].

A further gap concerns TFLN itself. Almost all existing studies of TFLN modulators are oriented toward telecom applications, where the evaluation focuses on propagation loss, the half-wave voltage length product *V*_π_*L*, and modulation bandwidth, leaving the multiphysics behaviors under FOG-relevant operating conditions largely unaddressed [27]. A few recent studies have begun to apply multiphysics modeling to TFLN modulators, coupling the optical, electrical and thermal fields for telecom-oriented bandwidth and reliability analysis [28,29] and examining thermo-mechanical degradation under temperature-cycling stress [30]. These works, however, target communication performance and device reliability; none formulates the sensor-grade bias-stability budget required for a FOG or jointly resolves the piezoelectric–photoelastic and pyroelectric channels that set the phase bias. TFLN devices are not physically equivalent to their bulk counterparts from a multiphysics standpoint: thin-film LN is typically patterned into high-index-contrast ridge waveguides with stronger mode confinement and a smaller electrode-to-waveguide spacing, so that the electric-field, strain [31], and temperature distributions all couple more tightly to the guided-mode effective index [32]. This concentrated geometry is doubly consequential—it boosts electro-optic efficiency and lowers the drive voltage, but it may simultaneously amplify the influence of piezoelectrically induced strain and local thermal gradients on the phase response. Consequently, transferring empirical conclusions from bulk LN devices to TFLN under FOG operating conditions tends to misrepresent the actual physics, and a Pockels-only analysis is insufficient to meet the environmental-robustness and phase-stability requirements of FOG-grade design.

Motivated by these considerations, the present work targets FOG sensing applications and develops a multiphysics analysis framework for an X-cut LNOI ridge phase modulator that self-consistently couples the electro-optic, piezoelectric–photoelastic, thermo-optic, and pyroelectric channels. A non-resonant phase-modulator architecture is adopted because the interferometric FOG requires a broadband, low-coherence source; high-Q resonant modulators, while compact and power-efficient, are narrowband and strongly temperature- and wavelength-sensitive, which is incompatible with broadband interferometric sensing and with the bias-stability objective of this work. This assessment is consistent with recent reviews of interferometric [33] and resonant [34] fiber-optic gyroscopes, and of integrated and planar photonic gyroscopes more broadly [35,36], which report that interferometric architectures currently lead in demonstrated accuracy, long-term bias stability, and engineering maturity. A finite-element implementation is used to quantitatively resolve the effective-index change and phase response of the device under realistic FOG operating conditions. Building on this framework, the slab thickness, ridge-top width, and electrode gap are systematically optimized against the requirements of sensor-grade operation, with the aim of balancing modulation efficiency, fabrication feasibility, and environmental robustness. The results provide quantitative design guidelines and an optimization pathway for compact, low-voltage, high-stability TFLN phase modulators suited to medium- and high-precision FOG systems.

The main contributions of this work are summarized as follows:

• A multiphysics model of a TFLN ridge phase modulator is developed that couples electro-optic, piezoelectric–photoelastic, thermo-optic, and pyroelectric effects within a unified finite-element framework.
• The individual contributions of the EO, PE (V-synchronous and V-independent), and TO/pyroelectric mechanisms are quantitatively decomposed; the analysis reveals that V-independent thermal-elastic PE reaches unignorable ~27% of the Pockels modulation depth at ΔT = 60 K, and that EO-only analysis systematically underestimates the bias-stability budget required for sensor-grade operation.
• The influence of slab thickness, ridge-top width, and electrode gap on the overlap factor and *V*_π_*L* is systematically investigated, yielding an optimized geometry with a push–pull *V*_π_*L* of 1.65 V·cm at a 4.4 μm electrode gap (25 °C) for sensor-grade operation, with the optimal geometry remaining stable across the 25~85 °C FOG operating range.
• The implications of the optimized design for FOG integration are discussed in terms of modulation efficiency, bias stability, and environmental robustness.
• A head-to-head comparison with competing phase-modulator platforms highlights the competitive advantages of TFLN for compact, stable, and low-power FOGs.

To the best of our knowledge, this is the first study that simultaneously couples all three channels and quantitatively decomposes their contributions in a FOG context.

## 2. Multiphysics Model for FOG-Oriented TFLN Modulators

### 2.1. Device Geometry

The proposed device is a ridge-type TFLN phase modulator designed for FOG applications. The waveguide is defined on an X-cut LNOI platform (thickness of wafer parameters from NANOLN) with light propagating along the Y-direction, so that the in-plane component of the applied electric field acts through the largest EO tensor element of LN. A coplanar electrode configuration generates the lateral modulation field across the ridge, and the ridge geometry is chosen to provide strong optical confinement while maintaining moderate fabrication complexity and good overlap between the guided mode and the applied field.

**Figure 1:**
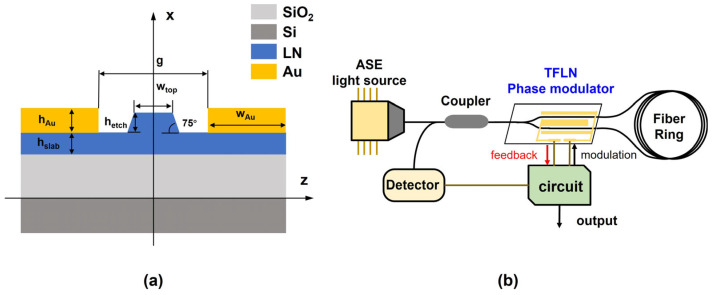
(**a**) Cross section schematic of the TFLN ridge phase modulator for FOG applications, showing the ridge waveguide, slab layer, coplanar electrodes, and the coordinate system; the *z* axis refers to the LN c-axis. (**b**) System schematic of the reflective FOG incorporating a TFLN phase modulator.

Three geometric parameters dominate the device behavior: the original TFLN slab thickness *h*_slab_= 100 nm, the ridge-top width *w*_top_ = 1.3 μm, the etching angle, which is set to 75° based on manufacturing experience, and the electrode gap *g* = 5 μm. As discussed later, these parameters jointly control the optical mode confinement, the electric-field distribution, the piezoelectric strain pattern, and the fraction of heat dissipated through the ridge. A parametric sweep of these three dimensions is therefore applied to identify a design that balances modulation performance, fabrication feasibility, and robustness against thermal and mechanical perturbations. The device cross-section and the reflective FOG system incorporating the phase modulator are illustrated in Figure 1a and Figure 1b, respectively.

### 2.2. Material Parameters and Boundary Conditions

**Figure 2:**
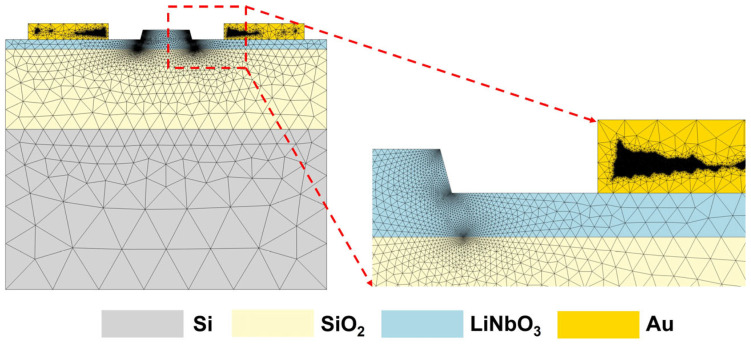
Finite-element mesh discretization at two zoom levels: (**left**) full cross section; (**right**) close-up of the LN ridge with electrode-corner refinement used to resolve field singularities, and the electrode-corner region showing the highest mesh density used to resolve field singularities. Mesh element sizes are ~2.5 nm at electrode corners and ~10 nm at the LN ridge and slab.

The optical, mechanical, piezoelectric, photoelastic, and thermal properties of each material layer are taken from established literature values, and summarized in Table 1. For the electrostatic problem, the two electrodes are set to a prescribed voltage difference and the surrounding domains are treated as electrically neutral dielectrics. For the mechanical problem, the bottom surface is clamped to represent the substrate support. For the thermal problem, the bottom boundary is held at a prescribed temperature (used both to emulate substrate heat-sinking and to impose controlled temperature rises), while the remaining external boundaries are treated as thermally insulating, unless otherwise stated. The fundamental quasi-TE mode is selected as the operating mode because of its strong overlap with the dominant *γ*_33_ element of the X-cut LN EO tensor.The finite-element mesh used in the simulation is shown in Figure 2, with element sizes of ~2.5 nm at electrode corners and ~10 nm at the LN ridge and slab to adequately resolve the field singularities.

For LiNbO_3_, the Pockels coefficients are *γ*_33_ = 30.9 pm/V and *γ*_13_ = 9.6 pm/V; the photoelastic constants (Weis and Gaylord, 1985 [17]) are *p*_11_ = −0.026, *p*_12_ = 0.090, *p*_13_ = 0.133, *p*_33_ = 0.071, and *p*_44_ = −0.075; the pyroelectric coefficient is *p*_3_ = −83 μC/(m^2^·K). The piezoelectric tensor dᵢⱼ uses the standard congruent-LN values from the following Refs.: [17] Weis and Gaylord, J. Appl. Phys. 1985; [37] Palik, Handbook of Optical Constants 1985; [38] Green, Sol. Energy Mater. 2008; [39] Johnson and Christy, Phys. Rev. B 1972.

### 2.3. Multiphysics Coupling Model

**Figure 3:**
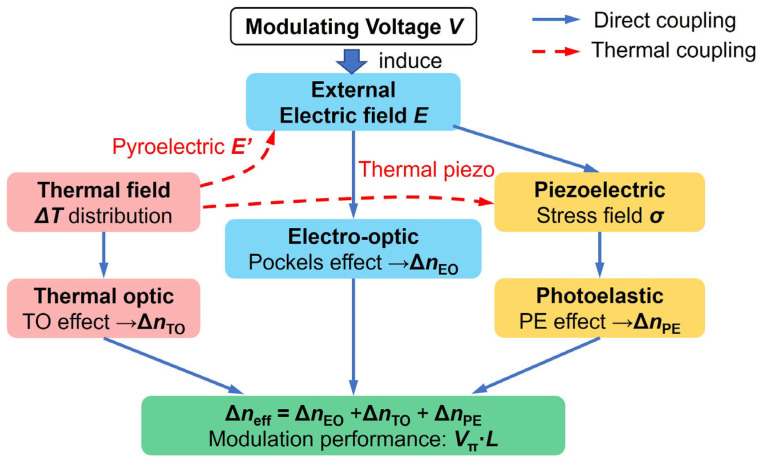
Multiphysics coupling framework, showing the four physical channels (electro-optic, photoelastic, thermo-optic, pyroelectric) and their direct (blue, solid) and thermal (red, dashed) interconnections. The total effective-index change Δ*n*_eff_ aggregates EO, PE, pyroelectric, and TO contributions and yields the composite half-wave voltage length product *V*_π_*L*.

A coupled multiphysics model is established by the FEM Multiphysics module that simultaneously solves an electrostatics module, a solid-mechanics module, a heat-transfer module, and an electromagnetic-wave mode solver, as schematically illustrated in Figure 3. The four modules are linked through the piezoelectric, photoelastic, thermo-optic, and pyroelectric constitutive relations of LiNbO_3_, so that field, strain, temperature, and polarization feed self-consistently into the optical mode problem.

The electro-optic, thermo-optic, and photoelastic responses of LiNbO_3_ are anisotropic and affect the extraordinary (*n*_e_) and ordinary (*n*_o_) indices differently; because the device operates on the quasi-TE mode (*E*‖*c*), the ne-relevant coefficients are used throughout. When an external voltage is applied to the electrodes, the electric field inside the LN ridge induces both a direct electro-optic (Pockels) index change [40] and, through the piezoelectric tensor, a mechanical strain in the waveguide; the strain, in turn, modifies the index through the photoelastic tensor. A temperature variation imposed from the substrate or from self-heating further modifies the index through the thermo-optic coefficient and, additionally, drives a quasi-static internal field through the pyroelectric coupling of LN. The total mode-averaged refractive-index perturbation seen by the guided mode can therefore be decomposed as

(1) $$\Delta n_{eff}=\Delta n_{EO}+\Delta n_{PE}+\Delta n_{TO}+\Delta n_{pyro}$$

where Δ*n*_EO_, Δ*n*_PE_, Δ*n*_TO_, and Δ*n*_pyro_ denote the electro-optic (Pockels), photoelastic (piezoelectrically induced), thermo-optic, and pyroelectric contributions, respectively. Each term is derived from the corresponding constitutive equation in the following subsections.

#### 2.3.1. Electro-Optic (Pockels) Contribution

The Pockels effect modifies the optical impermeability tensor through the linear electro-optic coefficients *γ*_ik_:

(2) $${\Delta (1/n^{2})}_{i}=\sum k\cdot \gamma_{ik}\cdot E_{k},i=1\ldots 6,k=1,2,3$$

For LiNbO_3_ (point group 3m), only four *γ*_ik_ elements are independent (*γ*_13_, *γ*_33_, *γ*_22_, *γ*_51_). In this X-cut configuration, the optic c-axis lies in-plane, transverse to the propagation direction (light propagates along Y), and the lateral coplanar-electrode field is predominantly *E*_x_, parallel to this c-axis. The TE-polarized mode (*E*‖c-axis) couples to the electric field through the dominant *γ*_33_ coefficient, yielding

(3) $$\Delta n_{EO,TE}\left(x,y\right)=-(1/2)\cdot n_{e}^{3}\cdot \gamma_{33}{\cdot E}_{x}(x,y)$$

where *n*_e_ is the extraordinary refractive index. Contributions from *γ*_13_ (TM coupling) and from the smaller *γ*_22_, *γ*_51_ elements are included in the simulation through the full tensor implementation in FEM implementation, but are sub-dominant for the TE operating mode and are suppressed by polarization filtering at the device input.

#### 2.3.2. Photoelastic (Piezoelectrically Induced) Contribution

The same applied voltage drives a strain field through the converse piezoelectric effect, with d*k*_q_ the piezoelectric strain coefficients of LN.

(4) $$\varepsilon_{q}=\sum_{k}dk_{q}\cdot E_{k},q=1,2\ldots 6$$

The resulting strain modifies the impermeability through the photoelastic tensor *p*_ij_:

(5) $${\Delta (1/n^{2})}_{i}\;=\;\sum_{j}p_{ij}\;\varepsilon_{j}\;,\;\;\;\;i,\;j\;=\;1,2\ldots 6$$

For 3m symmetry, *p*_ij_ reduces to eight independent constants (*p*_11_, *p*_12_, *p*_13_, *p*_14_, *p*_31_, *p*_33_, *p*_41_, *p*_44_).

Projecting Equation (4) onto the *n*_e_ index relevant for the TE mode in X-cut LN gives as follows:

(6) $$\Delta n_{PE,TE}(x,y)=-(1/2)n_{e}^{3}\cdot [p_{33}\varepsilon_{xx}+p_{13}\varepsilon_{yy}+p_{13}\varepsilon_{zz}]$$

where the laboratory frame (*x*, *y*, *z*) follows the device cross section of Figure 1, with *x* along the c-axis, which lies transverse to the propagation direction. All shear-strain contributions vanish identically for the ne index, because the relevant photoelastic elements *p*_3j_ with j = 4, 5, 6 are zero in the 3m symmetry-reduced photoelastic matrix. Equation (6) is implemented as a postprocessing variable *Dn*_PE,TE_ in simulation, and integrated against the optical mode density to obtain the mode-averaged Δ*n*_PE_.

#### 2.3.3. Thermo-Optic and Pyroelectric Contributions

There is a temperature rise Δ*T* relative to the reference state from *T*_ref_ = 25 °C to modify the refractive index directly through the thermo-optic coefficient, as

(7) $$\Delta n_{TO}(x,y)=(dn_{e}/dT)\cdot [T(x,y)-T_{ref}]$$

with d*n*_e_/d*T* = 3.3 × 10^−5^ K^−1^ for the TE mode in congruent LN. In addition, since LiNbO_3_ is pyroelectric, Δ*T* generates a polarization change Δ*p*_3_ = *p*_3_·Δ*T* along the c-axis (*p*_3_ = −83 μC m^−2^ K^−1^), which, under realistic grounded-electrode boundary conditions, establishes a quasi-static internal field *E*_pyro_ that re-enters the Pockels term. The pyroelectric contribution is therefore implicitly included in Δ*n*_EO_ through the self-consistent electrostatic solve at non-zero Δ*T*, and is reported separately as $\Delta n_{pyro}=-(1/2)n_{e}^{3}\cdot \gamma_{33}E_{pyro}$, *x* for diagnostic purposes, in Section 3.4.

#### 2.3.4. Mode-Weighted Overlap Factor and Figures of Merit

To connect the local index perturbations of Equations (3), (5) and (6) with the terminal phase response, we define a dimensionless electro-optic overlap factor $\Gamma_{EO}$ that captures the fraction of the electrode field converted into a useful Pockels perturbation of the optical mode:

(8) $$\Gamma_{EO}=g\cdot \int \Omega_{LN}Wopt(x,y)\cdot E_{x}(x,y)dA/[V_{0}\cdot \int \Omega_{all}W_{opt}(x,y)dA]$$

where *Ω*_LN_ is the LN core, *Ω*_all_ is the entire cross section, *ω*_opt_ is the optical energy density, *V*_0_ is the applied voltage, *E_x_* is the lateral electrostatic field across the electrode gap *g*, and the integrations span the LN core (numerator) and the full simulation domain (denominator). Defined in this way, $\Gamma_{EO}$ is unitless and is bounded between 0 and 1. Substituting Equation (3) into the mode integral yields the mode-weighted index change $\Delta n_{eff}\left(V_{0}\right)=-(1/2)n_{e}^{3}\cdot \gamma_{33}\Gamma_{EO}V_{0}/g$.

For the Y-branch push–pull configuration of the device, the two arms accumulate index changes of the opposite sign, doubling the differential phase, so the half-wave voltage-length product follows as

(9) $$V_{\pi }\cdot L=\lambda \cdot g/(2\cdot \Gamma_{EO}\cdot n_{e}^{3}\cdot \gamma_{33})$$

where λ is the operating wavelength; a smaller *V*_π_*L* indicates higher modulation efficiency. The factor of 2 in the denominator arises from the Y-branch push–pull configuration: the two arms accumulate Δ*ϕ* and −Δ*ϕ,* correspondingly, while doubling the differential phase swing for a given drive voltage relative to a single-arm modulator. The corresponding single-arm DC reference, recovered by removing the push–pull factor, is 2 $V_{\pi }\cdot L$. In the reflective FOG architecture used in this work (Figure 1), the optical signal traverses the modulator twice per measurement cycle (forward into the sensing coil and back), so the system-level half-wave voltage seen by the FOG electronics is half of the device-level $V_{\pi }\cdot L$ reported here. Finally, the overall phase shift accumulated along an interaction length *L* is

(10) $$\Delta \phi =(2\pi /\lambda )\cdot \Delta n_{eff}\cdot L$$

Equations (1)–(10) define the multiphysics-aware figure-of-merit framework used in Section 3 to decompose the simulated phase response of the optimized device.

The electrodes are assigned the complex refractive index of gold ( ${\tilde{n}}_{Au}$ = 0.55 + 11.5i at 1550 nm [39]), and the dielectric layers (LiNbO_3_, SiO_2_, Si) are treated as transparent. Consequently, the computed loss is electrode-induced ohmic absorption only. The complex mode solver yields *n*_eff_ = *n*′ + *iκ*, and the modal power-attenuation coefficient is derived as follows: $P(z)\propto exp(-2k_{0}\kappa z)$.

(11) $$\alpha [dB/cm]=8.686\cdot k_{0}\cdot Im(n_{eff})$$

where 8.686 = 20/ln10 (factor 2 means power vs. amplitude).

As the electrode gap decreases, the modal overlap with the metal increases, resulting in an increase in α from ≈0.26 dB/cm at *g* = 4.4 µm to >200 dB/cm below *g* = 2 µm.

### 2.4. Mode Tracking and Numerical Implementation

The fundamental quasi-TE mode of the X-cut ridge waveguide was solved with a full-vectorial finite-element mode solver coupled to the electrostatic and thermo-mechanical fields. At every geometry and drive voltage, the target mode was identified automatically as the eigenmode with optical confinement factor *Γ*_opt_ > 0.5, quasi-TE polarization purity > 0.7, and the highest effective index; this criterion tracks the same physical mode consistently across the full (*h*_slab_, *w*_top_, *g*) design space and at both 25 °C and 85 °C. The mesh was non-uniform and refined in the ridge core and the electrode-gap region, with the element density increased until the computed effective index and *V*_π_*L* were insensitive to further refinement.

## 3. Results and Discussion

### 3.1. Optical Mode Confinement

The fundamental quasi-TE mode of the X-cut ridge waveguide is first computed to establish the modal confinement and its overlap with the active modulation region. The optical field is predominantly confined within the LN ridge, with a moderate evanescent tail extending into the slab layer. This distribution is favorable for phase modulation because it concentrates the mode in the region where the lateral electric field, the piezoelectric strain, and the thermo-optic perturbation are all largest, while still retaining a finite tail that is insensitive to sidewall-roughness scattering. The optical field is the full-vectorial guided eigenmode of the ridge waveguide (not a Gaussian approximation); all overlap integrals use the true modal energy density.

**Figure 4:**
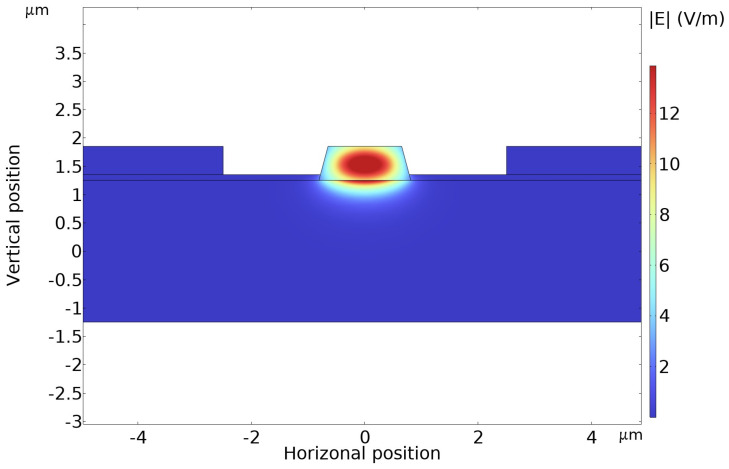
Fundamental quasi-TE optical mode distribution (*E*_x_ component, λ = 1550 nm) in the TFLN ridge waveguide baseline geometry before optimization with slab thickness 100 nm, ridge-top width 1.3 μm and electrode gap 5 μm.

The modal overlap is most sensitive to the ridge-top width and the slab thickness: narrower ridges improve the overlap with the lateral field but reduce fabrication tolerance, while wider ridges give stronger confinement at the cost of modulation efficiency. The geometry is therefore chosen to balance this trade-off, and its optimization is revisited quantitatively in Section 3.6. The fundamental quasi-TE mode field distribution (E_x component at λ = 1550 nm) for the baseline geometry is shown in Figure 4.

### 3.2. Electro-Optic Phase Response

**Figure 5:**
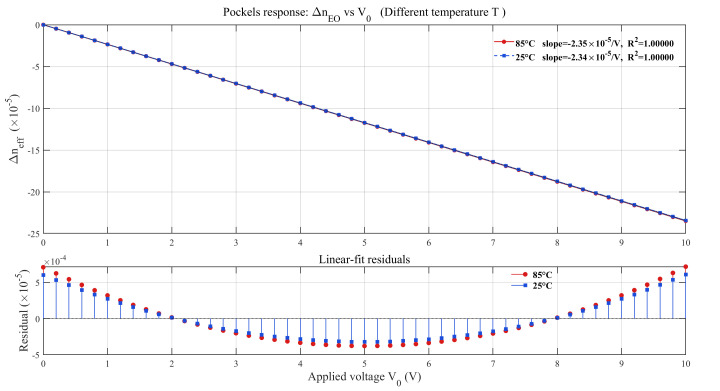
The effective-index shift and the overlap factor are presented as functions of applied voltage at temperatures of 25 °C and 85 °C. The baseline geometry is defined as *h*_slab_ = 100 nm, *w*_top_ = 1.3 μm, and *g* = 5 μm.

To isolate the intrinsic EO response, the PE and TO channels are temporarily disabled in the model. Under an applied voltage, the linear Pockels effect produces a refractive-index change that scales approximately linearly with voltage [41], consistent with the intrinsic response of LN. At the baseline geometry (gap = 5 μm), the simulation yields an electro-optic overlap factor *Γ*_EO_ = 0.540, corresponding to *V*_π_*L* = 1.70 V·cm (push–pull single-pass; equivalent to a system-level 0.85 V·cm in reflective FOG operation). The voltage dependence of Δ*n*_eff_ is highly linear over the *V*_0_ ∈ [0, 10] V range, with slope dΔ*n*/d *V*_0_ = −2.34 × 10^−5^ *V*^−1^ and *R*^2^ > 0.9999 at the baseline geometry, confirming the chirp-free Pockels response required for interferometric sensing, shown in Figure 5.

The approximate linearity of the difference between Δ*n* and the *V* relationship is precisely the behavior sought for interferometric sensing, where phase linearity directly determines scale-factor linearity. It has been demonstrated that, among the three geometric knobs, the electrode gap exerts the most direct influence on the local field strength. Reducing the gap increases the field and, consequently, the EO response. However, this reduction also tightens fabrication tolerance and may raise the risk of field-induced optical absorption. This trade-off is quantitatively analyzed in Section 3.6.

### 3.3. Piezoelectric-Strain and Photoelastic Response

**Figure 6:**
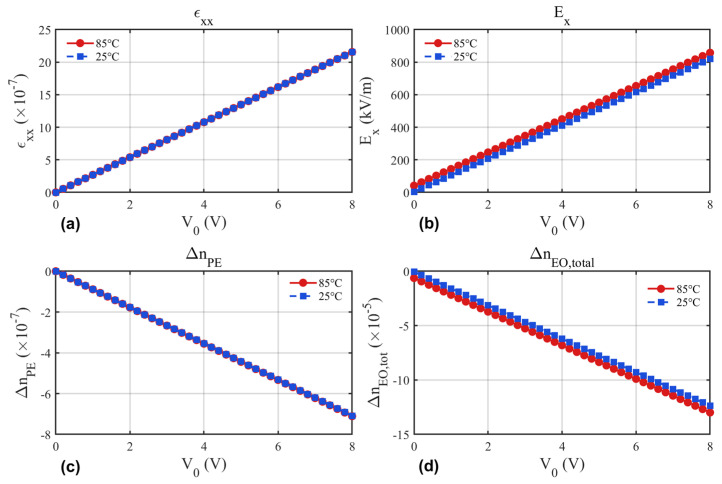
Piezoelectrically induced strain components under applied voltage and the corresponding photoelastic index perturbation. (**a**) Dominant strain component *ε*_xx_ vs. *V*_0_; (**b**) lateral electric field *E*_x_ vs. *V*_0_. (intercept at *V*_0_. = 0 reveals pyroelectric residual field at 85 °C); (**c**) mode-averaged photoelastic index shift Δ*n*_PE_ vs. *V*_0_; (**d**) total EO + pyroelectric index shift Δ*n*_EO,tot_ vs. *V*_0_. Red/blue lines refer to different temperatures: 85 °C/25 °C.

Re-enabling the piezoelectric coupling reveals the fact that the applied field generates a strain field in the LN layer through the converse piezoelectric effect. As shown in Figure 6a, the dominant strain component *ε*_xx_ scales linearly with *V*_0_ at a rate of approximately 2.69 × 10^−7^ V^−1^, with a near-zero intercept at both 25 °C and 85 °C, confirming that the voltage-driven converse-piezoelectric mechanism dominates the strain response.

The resulting photoelastic index perturbation Δ*n*_PE_, obtained by projecting the strain tensor through the photoelastic coefficients of LN and integrating over the optical mode, likewise exhibits a linear voltage dependence [Figure 6c], with a slope of approximately −8.9 × 10^−8^ *V*^−1^ and a negligible intercept at *V*_0_ = 0. At *V*_0_ = 5 V, the mode-averaged photoelastic contribution reaches Δ*n*_PE_ ≈ −4.5 × 10^−7^, which is only ~0.4% of the pure Pockels term Δ*n*_EO_ ≈ −1.17 × 10^−4^ at the same drive voltage. The PE contribution is therefore synchronous with the modulation signal, but more than two orders of magnitude weaker, producing a sub-percent correction to *V*_π_*L* that lies within typical fabrication tolerances.

Notably, the PE slope is virtually identical at 25 °C and 85 °C (−8.86 × 10^−8^ vs. −8.89 × 10^−8^ V^−1^), indicating that the photoelastic channel is thermally insensitive across the FOG operating temperature range. This thermal invariance simplifies the multiphysics design: the PE channel tracks the drive signal with a fixed, small scaling factor and does not introduce additional temperature-dependent bias drift.

As illustrated in Figure 6b, the lateral electric field *E*_x_ is depicted as a function of *V*_0_. At an operating temperature of 85 °C, a non-zero intercept of approximately 4.2 × 10^4^ V/m is observed at *V*_0_ = 0, while at 25 °C, the intercept approaches 3.2 × 10^3^ V/m. This residual field has been traced to the pyroelectric polarization charge of LN under thermal load, and a thorough analysis of this phenomenon can be found in Section 3.4. As illustrated in Figure 6d, the pyroelectric index offset is evident as a downward shift in the 85 °C Δ*n*_EO,tot_ curve, relative to the 25 °C curve at *V*_0_ = 0, amounting to a change in the pyroelectric index of approximately −6.3 × 10^−6^ at an applied temperature of 85 °C. This shift corresponds to approximately 27% (mode-field weighted; equivalent to 5.4% in spatial average) of the Pockels modulation depth at an applied voltage of *V*_0_ = 5 V.

From the perspective of a sensing application, the PE contribution is of particular significance, due to its inherent connection to the electrical drive. Each modulation cycle imprints a synchronous strain cycle on the waveguide [42], and any external vibration, packaging stress, or residual fabrication stress couples into the same photoelastic channel. The incorporation of the PE term consequently leads to alterations in both the predicted modulation efficiency and, more significantly, the predicted sensitivity of the phase bias to mechanical perturbations. This quantity exerts a direct influence on the stability of FOG bias.

### 3.4. Thermo-Optic and Pyroelectric Response Under Thermal Load

**Figure 7:**
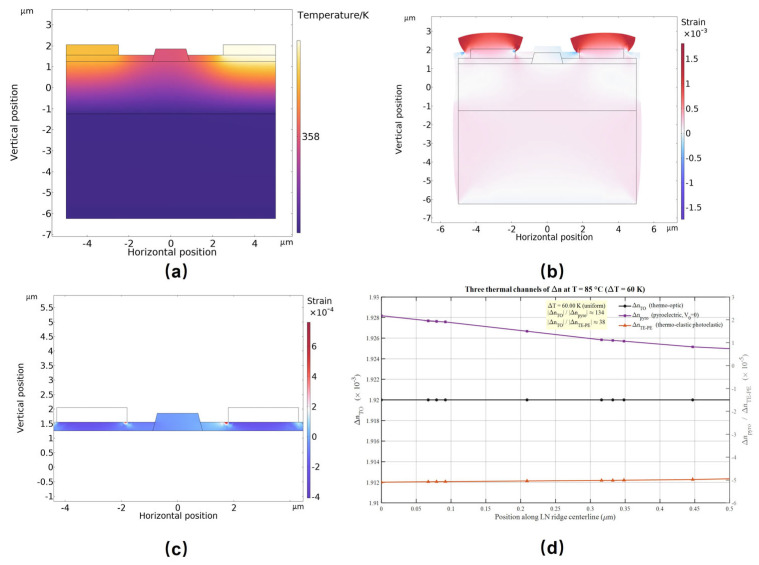
Temperature-field distribution under substrate heating and the corresponding thermo-optic refractive-index shift. (**a**) Steady-state temperature field at 85 °C (Δ*T* = 60 K). (**b**) Dominant strain component *ε*_xx_ distribution. (**c**) Lateral thermal–photoelastic-induced strain distribution. (**d**) Spatial profiles of the three thermal refractive-index perturbations along the ridge centerline: thermo-optic Δ*n*_TO_ (black), pyroelectric Δ*n*_pyro_ (purple), and thermo-elastic photoelastic Δ*n*_PE,thermal_ (orange).

Under elevated substrate temperature (the temperature of the bottom boundary of the Si substrate is set to be 358.15 K, room ambient), the simulated temperature field is laterally non-uniform across the device cross section. Under elevated operating temperature *T*_op_ = 358.15 K and reference temperature *T*_ref_ 298.15 K, the temperature change Δ*T* = 60 K, the simulated temperature field reaches steady state with the LN ridge equilibrated near Top, with a small residual gradient (<5 K) within the LN laye (shown in Figure 7a). The mode-weighted Δ*T* seen by the optical mode is therefore close to the full 60 K excursion.

Three temperature-driven channels are identified that perturb the effective refractive index:

(i) Direct thermo-optic response: Δ*n*_TO_ = (d*n*_e_/d*T*) × Δ*T*_LN_ ≈ 1.9 × 10^−3^ (averaged over the LN ridge), acting as a common-mode phase shift that cancels in the differential modulator branch output.
(ii) Pyroelectric-induced electro-optic response: spontaneous polarization of LN under Δ*T* generates a surface charge density *σ* = *p*_pyro_·Δ*T*, which would establish an internal field driving *γ*_33_ in the absence of applied voltage. The pyroelectric origin of this contribution, in conjunction with its comparatively benign behavior in X-cut films—in contrast to Z-cut, where the same effect produces long-lived refractive-index drift—has been directly observed in high-Q TFLN micro resonators [43]. This observation supports both the magnitude adopted here and the choice of an X-cut platform for bias-stable operation. Under realistic operating bias (one electrode driven, the other grounded), the bulk of this pyroelectric charge is drained through the external circuit, leaving only a residual mode-averaged field of ~4.2 × 10^4^ V/m within the LN at *V*_0_ = 0. The corresponding pyroelectric-induced index shift is Δ*n*_pyro_ = −6.3 × 10^−6^ at Δ*T* = 60 K, voltage-independent across the 0~10 V drive range, and approximately 27% (mode-field weighted; equivalently 5.4% in spatial average) of the Pockels modulation depth at 5 V applied voltage. To confirm the pyroelectric origin, the same simulation was repeated at the room-temperature operating point (*T* = 25 °C, Δ*T* ≈ 5 K above the reference state), yielding *E*_x_ ≈ 3.2 × 10^3^ V/m and Δ*n*_pyro_ = −4.8 × 10^−7^. This value is approximately 13× smaller than the 85 °C result, and within approximately only 0.06% of the 5 V applied voltage Pockels modulation depth. The 13× scaling between the two operating points is consistent with the linear Δ*P* = *p*_3_·Δ*T* relation expected for the pyroelectric mechanism, ruling out artefactual contributions from the meshing or solver and confirming that the residual is genuinely thermal–electrical in origin and effectively negligible at room temperature.
(iii) Thermo-elastic photoelastic response: the substrate-clamped thermal expansion of LN produces strain ε ≈ α·Δ*T* throughout the ridge, which is then converted to an index bias through the photoelastic tensor (shown in Figure 7b,c). The thermal strain is partially relieved by the bonded substrate stack and the integrated photoelastic response is opposite-signed to the pyroelectric channel. The combined V-independent residual measured by our coupled simulation is Δ*n*_thermal_ = −6.3 × 10^−6^ at Δ*T* = 60 K/*V*_0_ = 0, encompassing both pyroelectric and thermo-elastic photoelastic contributions. This is bounded to ~27% (mode-field weighted) or ~5.4% (spatially-averaged) of the Pockels modulation depth at 5 V applied voltage, two orders of magnitude smaller than worst-case floating-electrode estimates of ~10^−3^.

The spatial profiles of the three thermal refractive-index perturbations (Δ*n*_TO_, Δ*n*_pyro_, and Δ*n*_PE,thermal_) along the ridge centerline are compared in Figure 7d. Crucially, although these temperature-driven contributions are large, they all behave as static biases that do not scale with *V*_0_. The pure-electrical *V*_π_*L* therefore remains stable to within 0.3% across the 60 K range. What does drift, however, is the MZI bias point:

Δϕ_bias_/L = (2π/*λ*) × Δ*n*_thermal_ ≈ 0.26 rad/(cm × Δ*T* = 60 K) (Δ*n*_TO_ is common-mode and cancels in MZI output).

In a 1 cm modulator, this corresponds to a 0.08π (≈0.04 fringe) bias swing across the operating temperature range—a quantity that a FOG must compensate via servo loop. We emphasize, however, that the V-independent thermal channels (pyroelectric and thermo-elastic photoelastic) are absent from Pockels-only modeling, and must be included for accurate bias-stability prediction in sensor-grade design, even when their absolute magnitude is modest. Translated into a drive-voltage equivalent through the measured Pockels slope (dΔ*n*_EO_/d*V*_0_ ≈ −2.34 × 10^−5^ V^−1^), the *V*_0_ = 0 thermal residual at Δ*T* = 60 K corresponds to a DC offset of |Δ*n*_thermal_/(dΔ*n*_EO_/d *V*_0_)| ≈ 0.27 V; the corresponding offset at ΔT ≈ 5 K above the reference state (*T* = 25 °C) is only ~0.021 V. This sub-volt, slowly-varying offset is the quantity that a temperature-compensation electronics path must track to keep the FOG read-out locked to its *V*_0_ = 0 quadrature point, and is well within the standard ±5 V drive range of FOG-grade phase-modulator electronics.

### 3.5. Multiphysics Decomposition of the Refractive-Index Change

**Figure 8:**
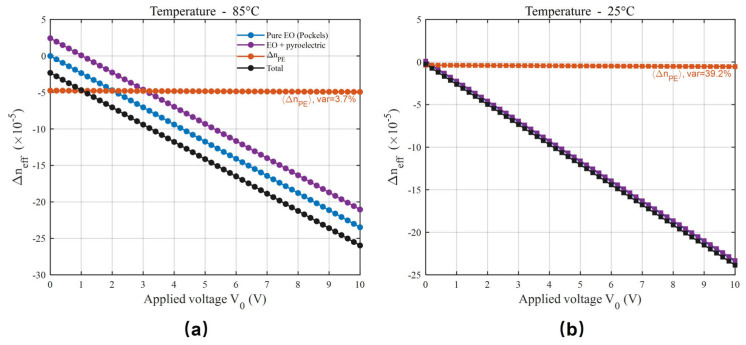
Multiphysics decomposition of the mode-averaged effective-index change at *V*_0_ = 5 V and Δ*T* = 60 K (85 °C): EO (Pockels), V-synchronous PE, and V-independent thermal (pyroelectric + thermo-elastic PE) contributions. Panels: (**a**) 85 °C, (**b**) 25 °C.

To assess the relative importance of the three mechanisms, the total effective-index change is decomposed into its EO, PE, and TO components under identical operating conditions. As summarized in Figure 8, the EO term dominates under normal drive, as expected from the strong Pockels response of LN. The PE and TO terms are smaller, but non-negligible, and, depending on the local strain and temperature distribution, can either reinforce or partially offset the EO contribution. Quantitatively, at *V*_0_ = 5 V and Δ*T* = 0, the Voltage-synchronous photoelastic contribution Δ*n*_PE,V-sync_ = −4.5 × 10^−7^ is only ~0.6% of the pure Pockels term Δ*n*_EO_ = −1.17 × 10^−4^. Under thermal load (ΔT = 60 K) with operating bias (one electrode-driven, the other grounded), the mode-field-weighted V-independent thermal residual Δ*n*_thermal,mode_ = −3.1 × 10^−5^ (or −6.3 × 10^−6^ in spatial average) is approximately 27% (or 5.4% spatially-averaged) of the modulation depth at *V*_0_= 5 V. Furthermore, comparing the pure Pockels slope at *T* = 25 °C and *T* = 85 °C reveals that the EO modulation efficiency itself is thermally stable to within 1.5% (the explicit 25 °C/85 °C FEM co-simulation reported in Figure 5 yields slopes of −2.342 × 10^−5^ V^−1^ and −2.343 × 10^−5^ V^−1^, respectively, differing by 0.01%, well within this bound), indicating that the dominant temperature-driven term is a V-independent bias offset, rather than a degradation of modulation efficiency. Pockels-only analysis therefore captures the modulation signal accurately to within ~10%, but misses the V-independent thermal- bias channel that determines bias drift in sensor-grade operation.

Two implications follow from this decomposition. Firstly, a model that retains only the Pockels term systematically mispredicts the magnitude of the phase response. For a FOG application, this results in calibration errors in the scale factor [44]. Secondly, the PE and TO channels are the ones that couple the modulator to its mechanical and thermal environment. Consequently, they are precisely the channels that determine bias drift and environmental sensitivity. Within the domain of evaluating a TFLN modulator for sensing applications, there arises a necessity for the incorporation of the aforementioned elements into the assessment framework.

### 3.6. Geometry Optimization for Sensor-Oriented Performance

By establishing the multiphysics model, the optimization of the three dominant geometric parameters is now underway: slab thickness *h*_slab_, ridge-top width *w*_top_, and electrode gap *g* are optimized to balance modulation efficiency and environmental robustness. The three parameters were examined independently, for as to how they pertained to the tracking of *Γ*, *V*_π_*L*, and the sensitivity of the phase response to the PE and TO channels.

Slab thickness mainly controls the modal confinement in the ridge [45,46,47]. Thinner slabs concentrate the field in the ridge region and improve Γ, but excessive thinning increases scattering loss, reduces fabrication tolerance, and aggravates the lateral thermal non-uniformity highlighted in Section 3.4. The optimum is found at *h*_slab_ = 300 nm, which corresponds to leaving the unetched LN slab at its as-deposited thickness on a 600 nm device-layer wafer (equivalent to a 300 nm ridge etch depth).

Ridge-top width sets the modal size and its overlap with the lateral field. Too narrow a ridge improves Γ but increases sidewall-scattering loss and tightens lithography tolerance; too wide a ridge reduces Γ and dilutes the modulation. The optimum lies at an intermediate value. Although the bare *V*_π_*L* exhibits a shallow local minimum at *w*_top_ ≈ 1.0 μm at 85 °C, this narrow-ridge regime is excluded from the operating window by single-mode constraints and lithography tolerance, both of which are tighter at sub-1.2 μm ridge widths in TFLN platforms [46].

**Figure 9:**
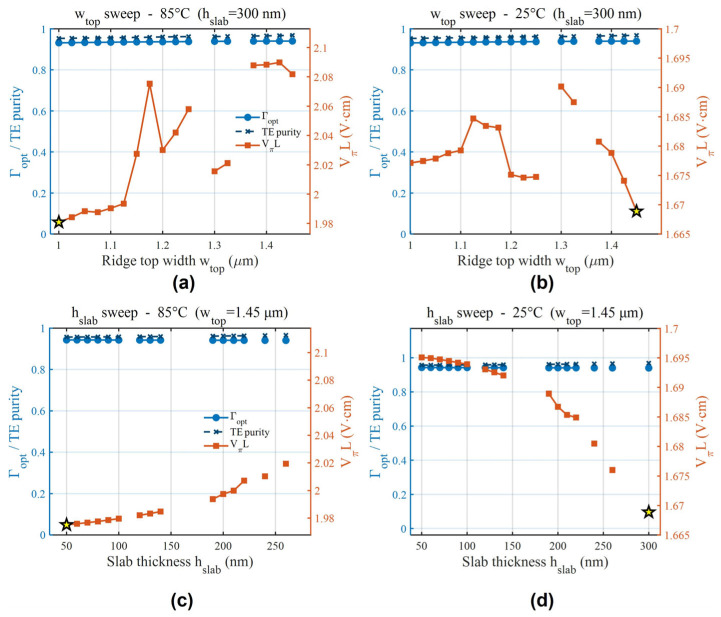
Geometric optimization of the TFLN ridge phase modulator. Ridge-top-width wtop sweep at (**a**) 85 °C and (**b**) 25 °C, with slab thickness fixed at *h*_slab_ = 300 nm. Slab-thickness *h*_slab_ sweep at (**c**) 85 °C and (**d**) 25 °C, with ridge-top width fixed at *w*_top_ = 1.45 μm. Electrode gap *g* = 5 μm. Left axis: optical confinement factor *Γ*_opt_ and TE purity; right axis: half-wave voltage-length product *V*_π_*L* (push–pull single-pass). Yellow stars mark the *V*_π_*L* minimum within the swept range; the selected operating point (*h*_slab_ = 300 nm, *w*_top_ = 1.45 μm) is determined jointly with the electrode-gap sweep in the subsequent figure.

Figure 9 summarizes the geometric optimization at both operating temperatures. The optical confinement factor *Γ*_opt_ and the TE purity remain essentially flat at 0.94 ± 0.01 over the entire (*w*_top_, *h*_slab_) sweep range at both 25 °C and 85 °C, confirming that the targeted quasi-TE_00_ mode is robustly tracked across the design space and that residual variations in *V*_π_*L* reflect genuine modulation-efficiency changes, rather than mode-tracking artifacts. Within the swept range, *V*_π_*L* varies in a narrow window (1.650~1.682 V·cm push–pull at 25 °C) and exhibits a shallow plateau spanning *w*_top_ ≈ 1.40~1.45 μm and *h*_slab_ ≈ 200~300 nm, within which the selected operating point (*h*_slab_ = 300 nm, *w*_top_ = 1.45 μm) lies. When *V*_π_*L* is extracted from the Pockels modulation slope, the 85 °C landscape is essentially unchanged (within ~0.3%), preserving the same plateau and optimum; the ~17% elevation seen with a single-voltage estimate is an artifact of the voltage-independent pyroelectric bias (Section 3.4), and is removed by the slope-based extraction.

**Figure 10:**
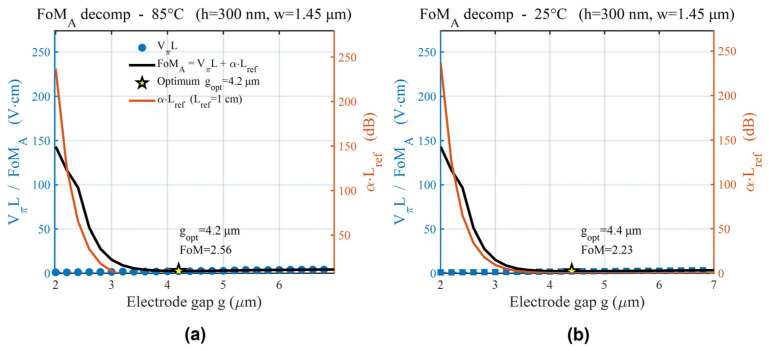
Electrode-gap optimization at the optimal waveguide cross section (*h*_slab_ = 300 nm, *w*_top_ = 1.45 μm). Panels (**a**) 85 °C and (**b**) 25 °C: half-wave voltage-length product *V*_π_*L* (push–pull, single-pass; blue dots, left axis), normalized propagation loss *α*·*L*_ref_ with *L*_ref_ = 1 cm (red curve, right axis), and the composite figure of merit FoMA = *V*_π_*L* + *α*·*L*_ref_ versus electrode gap. FoMA stays low and nearly flat for g ≳ 4 μm and rises steeply below ~3 μm; the selected gaps (4.4 μm at 25 °C, 4.2 μm at 85 °C) lie on the low-loss plateau, just above the loss knee.

Optimum at *h*_slab_ = 300 nm, *w*_top_ = 1.45 μm, *g* = 4.4 μm (temperature-independent; see Figure 10 for the FoMA vs. gap sweep at both temperatures) yields *V*_π_*L* = 1.65 V·cm (push–pull single-pass) with *Γ*_EO_ ≈ 0.545, optical confinement factor *Γ*_opt_ = 0.940, and propagation loss α ≈ 0.26 dB/cm at the 25 °C optimum. The half-wave voltage demonstrates a negligible dependence on the electrode gap within the practical range, exhibiting values of 1.70, 1.65, and 1.62 V·cm at *g* = 5.0, 4.4, and 2.8 μm (push–pull, single-pass), with a total variation of less than 5%. Conversely, the electrode-induced loss experiences a pronounced increase as the gap diminishes, with α approximating 0.08, 0.26, and 5.9 dB/cm at equivalent gaps. This increase exceeds 200 dB/cm below 2 μm. The composite figure of merit FoMA = *V*_π_*L* + *α*·*L*_ref_ (dB), *L*_ref_ = 1 cm, therefore remains low and nearly flat for *g* ≥ 4 μm (≈1.8–1.9) and diverges below ~3 μm (7.5 at 2.8 μm). We designate *g* = 4.4 μm as a representative point on this low-loss, low-*V*_π_*L* plateau, situated just above the knee, beyond which metal-induced absorption becomes prohibitive. The selection is essentially temperature-independent, because *V*_π_*L*, *γ*_33_, and *α* are all thermally stable across 25–85 °C. In this geometry, the optical confinement factor *Γ*_opt_ remains essentially unchanged from its baseline value (0.940 across the entire optimization range), confirming that the modal field stays well-localized in the LN ridge and that the *V*_π_*L* reduction comes purely from the increase in *Γ*_EO_ as the field approaches the LN core, not from any redistribution of the optical mode itself.

At an operating temperature of 85 °C, the optimal geometry and gap remain constant (*h*_slab_ = 300 nm, *w*_top_ = 1.45 μm, *g* = 4.4 μm). Additionally, the extracted *V*_π_*L*, derived from the Pockels modulation slope, maintains a constant value of 1.65 V·cm (push–pull single-pass), with a temperature-related stability of ±0.3%, consistent with the temperature-stable slopes observed in Section 3.5. At 85 °C, this includes the voltage-independent pyroelectric/thermo-elastic bias (approximately + 2.4 × 10^−5^, Section 3.4), which does not participate in modulation. The slope-based extraction removes it by construction (Figure 9). Consequently, the temperature sensitivity is attributed to the bias offset, rather than the half-wave voltage. The electrode gap controls the lateral field strength and, therefore, the EO response, most directly. Narrower gaps lower *V*_π_*L*, but raise the risk of optical absorption in the metal, and increase alignment sensitivity during fabrication. The chosen value of gap = 4.4 μm provides the best overall balance across the 25~85 °C operating range.

Compared with the initial design (gap = 5 μm), the optimized geometry reduces *V*_π_*L* by 3% (from 1.70 to 1.65 V·cm push–pull, single-pass),with the full performance comparison summarized in Table 2. Compared with the initial gap = 5 μm design, the optimized geometry trims *V*_π_*L* by 3% (1.70 to 1.65 V·cm), while keeping loss below ~0.3 dB/cm; the binding consideration is not a sharp efficiency optimum, but the avoidance of the steep loss penalty at sub-3 μm gaps. The PE and TO bias perturbations remain essentially unchanged across geometries because they are dominated by the LN material constants and the thermal field, not by the local geometry—a robustness property that simplifies multiphysics-aware design. The intrinsic absorption of LiNbO_3_ at 1550 nm is negligible, relative to the electrode-induced loss (the dielectric layers are lossless in Table 1), so the computed *α* (≈0.26 dB/cm) is electrode-induced only. It has been established that, within the tens-of-nanometer bandwidth of a broadband FOG source, the modal indices and coupling coefficients are effectively dispersionless. Given the proportionality of *V*_π_*L* to λ, the half-wave voltage varies by only ≈1–2% across the band, a phenomenon referred to as a common-mode effect.

**Table 2:** Device performance before and after optimization. All *V*_π_*L* values are reported under the push–pull single-pass convention used throughout this work; the corresponding system-level values in reflective FOG operation are half of the listed numbers.

Parameters	Origin Gap = 5 μm	Optimization Gap = 4.4 μm (@25 °C)	Improvement
*n*_eff_	1.9034	1.940	+0.4%
*Γ*_opt_	0.838	0.940	+12.2%
*Γ*_EO_	0.540	0.545	+0.9%
*V*π·*L*@ΔT = 60 K	1.70 V·cm	1.65 V·cm	−2.9%
Δ*n*_PE bias_@ΔT = 60 K	−4.5 × 10^−7^	−4.5 × 10^−7^	PE slope ratio~(0.4%)
Δ*ϕ*	0.084π rad/cm	0.083π rad/cm	<1% (geometry-independent)

### 3.7. Fabrication Tolerance

**Figure 11:**
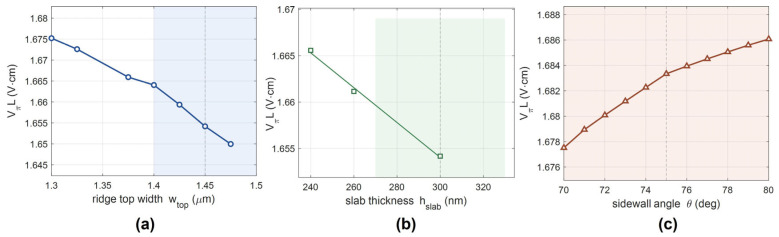
*V*_π_*L* sensitivity to fabrication variations at 25 °C (push–pull, Pockels-slope extraction): (**a**) ridge-top width *w*_top_, (**b**) slab thickness *h*_slab_, and (**c**) sidewall angle *θ*. Shaded bands mark realistic process windows (±50 nm, ±30 nm, ±5°); dashed lines mark the nominal values. *V*_π_*L* stays within ≈1% in every case.

To assess the system’s robustness against realistic process variation, a one-at-a-time sensitivity analysis was carried out at 25 °C around the optimized geometry (slab thickness 300 nm, ridge-top width 1.45 µm, electrode gap 4.4 µm, sidewall angle 75°) with *V*_π_*L* extracted from the Pockels modulation slope (Figure 11). The optimum is located on a shallow plateau: *V*_π_*L* varies by only ≈0.85% for a ±50 nm ridge-width deviation (see Figure 11a), ≈0.7% for a ±30 nm slab-thickness (etch-depth) deviation (see Figure 11b), and ≈ 0.5% for a ±5° sidewall-angle deviation (see Figure 11c), while the waveguide remains single-mode with optical confinement *Γ*_opt_ ≈ 0.94 and quasi-TE purity ≈ 0.96 throughout. The electrode gap is optimized separately in Section 3.6 (Figure 10); the selected 4.4 µm lies on the flat region of both the *V*_π_*L*–gap and loss–gap curves, so a ±0.3 µm gap tolerance is negligible. A misalignment between the rigid electrode and the waveguide has been shown to perturb *V*_π_*L* only at second order by symmetry (generally less than 0.5% for ±0.3 µm). The dominant effect of this misalignment is on the left/right loss balance and residual chirp, which lie beyond the scope of the present 2-D modal analysis. Sidewall-roughness scattering is a longitudinal effect that is not captured by the eigenmode model, and contributes to a slight propagation loss (typically 0.1–0.3 dB/cm). Packaging-induced stress couples through the same photoelastic channel that has already been modeled and, as a result, contributes to the temperature/stress bias budget, rather than to *V*_π_*L*. It is imperative to note that the photoelastic and pyroelectric bias residuals are governed by the LiNbO_3_ material constants and the temperature/stress field, rather than by local waveguide geometry. These residuals remain essentially constant across the fabrication windows. The design is therefore fabrication-tolerant, and the engineering value of the optimization lies in the multiphysics bias-stability budget, rather than in the approximate 3% reduction of *V*_π_*L*.

### 3.8. Comparison with Competing Platforms and Implications for FOG Integration

Table 3 compares the present device with representative phase-modulator platforms reported to date. Conventional Ti: LiNbO_3_ modulators remain the de facto FOG standard because of their linear Pockels response, but their centimeter-scale footprint and several-volt driving requirement are mismatched with the miniaturization trend. Silicon and InP modulators achieve attractive compactness, but either rely on a nonlinear plasma-dispersion effect (Si) or require active temperature stabilization (InP), both of which compromise sensing-grade phase fidelity. Prior TFLN phase modulators, while outstanding for communications, have not been characterized under the full electro-opto-mechano-thermal coupling that governs their use in FOGs. The present device inherits the compactness, linear Pockels response, and low loss of the TFLN platform, and, in addition, is the first, to the best of our knowledge, to be co-designed against the PE and TO channels that set the phase-bias stability inside a FOG.

Thin-film lithium tantalate (TFLT) has emerged as a promising alternative, exhibiting comparable *γ*_33_ (approximately 30 pm/V, hence similar *V*_π_*L*) with ≈17× lower birefringence, a higher optical-damage threshold, lower RF loss, and markedly improved DC bias stability (e.g., <1 dB bias drift over 46 h, versus ~5 dB for a comparable TFLN device) [49]. The lower birefringence is advantageous for polarization-critical FOGs, and the enhanced DC stability aligns with the bias-drift focus of this work. The primary trade-off is the comparatively lower maturity of the TFLT fabrication ecosystem relative to TFLN [50]. The present multiphysics framework applies directly through substitution of the corresponding material tensors.

## 4. Conclusions

**Figure 12:**
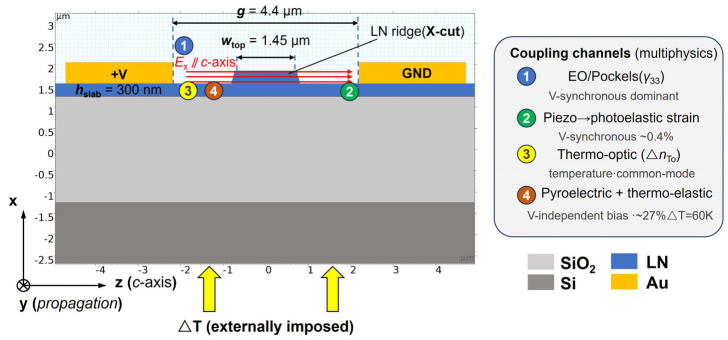
Schematic of the optimized modulator geometry (slab 300 nm, top width 1.45 µm, gap 4.4 µm, quasi-TE, E‖c) and its four coupling channels.

Figure 12 summarizes the resulting sensor-optimized configuration—an X-cut TFLN ridge (slab 300 nm, top width 1.45 µm, sidewall 75°) with coplanar electrodes at a 4.4 µm gap, operated in the quasi-TE (E‖c) mode, and the four coupling channels analyzed in this work—electro-optic (Pockels), piezoelectric–photoelastic, thermo-optic, and pyroelectric—are annotated, distinguishing the modulation-active Pockels term from the voltage-independent thermal-bias channels.

We have presented a unified multiphysics model of a thin-film lithium niobate ridge phase modulator designed for fiber-optic gyroscope operation. By self-consistently coupling the electro-optic, piezoelectric–photoelastic, and thermo-optic channels under realistic operating boundary conditions, we have shown that the V-driven response is dominated by the Pockels term, while V-independent thermal effects (pyroelectric and thermo-elastic photoelastic, the combined ~27% of the modulation depth at *V* = 5 V at Δ*T* = 60 K) act as bounded bias offsets that are routinely omitted from communication-oriented analyses, but must be quantified for sensor-grade design. Both the geometric EO overlap (*Γ*_EO_, stable to within 0.02%) and the Pockels-slope half-wave voltage (*V*_π_*L*, stable to within 0.3%) are thermally stable from 25 °C to 85 °C, so the modulation efficiency is essentially temperature-independent and the principal environmental concern is bias drift, rather than modulation-efficiency degradation. A systematic optimization of the slab thickness, ridge-top width, and electrode gap yields a device with *V*_π_*L* = 1.65 V·cm (push–pull single-pass) with quantified V-synchronous photoelastic and V-independent thermal residual contributions bounded to ~0.4% and ~27% (mode-field-weighted) of the modulation depth, respectively.

It is important to note that the V-synchronous PE component is thermally invariant, exhibiting a variation of approximately 1% between 25 °C and 85 °C. In contrast, the V-independent thermal–elastic PE component demonstrates a substantial increase, reaching approximately thirteenfold with an increase in temperature of 60 K. This phenomenon is only resolvable through coupled multiphysics analysis, and is not observed in telecom-oriented *V*_π_*L*-only characterizations.

Benchmarked against conventional bulk LN, silicon, InP, and prior communication-oriented TFLN modulators, the present device combines a compact footprint, a linear Pockels response, and, for the first time, an explicit multiphysics budget for environmental stability, positioning it as a promising phase-modulator platform for compact, low-power, and thermally robust FOGs. Future work will focus on experimental validation of the predicted multiphysics budget against fabricated devices, and on extending the framework to integrated polarization, maintaining interfaces required for full FOG chip-scale integration.

The constitutive framework is general: it extends to high-speed RF and telecom modulators by retaining the Pockels and overlap terms, treating the thermo-optic and pyroelectric channels as slow bias-drift contributions, and adding piezoelectric–acoustic resonance dynamics, together with traveling-wave electrode design, for velocity and impedance matching; the quasi-static weights reported here are specific to the FOG regime.

## Figures and Tables

**Table 1:** Material parameters used in the simulation.

Material	Thickness	*n* @ 1550 nm	*ε*_r_	d*n*/dT (10^−5^/K)	α (10^−6^/K)	Ref.
LiNbO_3_ (X-cut, congruent)	Total 600 nm (300 nm slab + 300 nm ridge)	*n*_e_ = 2.138, *n*_o_ = 2.211	*ε*_11_ = 43, *ε*_33_ = 28	d*n*_e_/dT = 3.3, d*n*_o_/dT = 0.6	α_a_ = 15.4, α_c_ = 7.5	[17]
SiO_2_ (BOX)	4.7 μm	1.444	3.9	1.0	0.55	[37]
Si (substrate)	400 μm	3.476 (transparent)	11.7	18.6	2.6	[38]
Au (electrode)	0.5 μm	0.55 + 11.5i (complex)	—	—	14.2	[39]

**Table 3:** Comparison of integrated phase-modulator platforms relevant to fiber-optic gyroscopes.

Platform	*V*_π_ (V)	*V*_π_*L* (V·cm)	Footprint	Thermal/Mechanical Analysis	Sensing Suitability
Bulk Ti: LiNbO_3_ (applied in conventional FOG [5])	3~5	≈10~15	cm scale	EO only; DC drift known but not co-modeled	Mature, but bulky and power-hungry
Silicon/SOI [7]	5~7	≈1~2	mm scale	EO only (plasma-dispersion, nonlinear)	Compact, but nonlinear response unsuitable for interferometric sensing
InP [8]	1.5~2	≈1~2	mm scale	EO only; TEC mandatory	Compact, but active cooling limits FOG integration
TFLN photonic crystal (resonant) [46]	n/a (res.)	tuning 1.98 GHz/V (≈16 pm/V); 0.58 µm^3^; 22 fJ	wavelength scale	EO only; high-Q resonance, sensitive to T/λ	Ultra-compact/low-energy, but narrowband → unsuitable for broadband FOG
TFLN MZM (telecom) [13,14]	1.4	≈2.3	mm scale (BW > 45 GHz)	EO only	High efficiency; sensing-grade stability not validated
x-cut TFLN phase modulator in I-FOG [48]	—	2.2	mm scale (10 mm)	EO only	Demonstrated in a FOG; stability not multiphysics-modeled
TFLT [49,50]	—	≈3.4	mm scale	EO (*γ*_33_ ≈ 30 pm/V); ≈17× lower birefringence; improved DC bias stability	Promising: best DC stability (<1 dB vs. 5 dB over 46 h); platform less mature than TFLN
**This work** **(TFLN, sensing-oriented)**	**1.65 V (@1 cm)**	**1.65**	**mm scale**	**EO + piezoelectric-PE + thermo-optic + pyroelectric coupled**	**Sensor-grade stability predicted by full multiphysics**

## Data Availability

The original contributions presented in this study are included in the article. Further inquiries can be directed to the corresponding author.
